# Bordering seafarers at sea and onshore

**DOI:** 10.3389/fsoc.2022.1084598

**Published:** 2023-01-24

**Authors:** Georgie Wemyss

**Affiliations:** Centre for Research on Migration, Refugees and Belonging (CMRB), University of East London, London, United Kingdom

**Keywords:** bordering, embodiment, seafarers, *lascar*, coloniality, maritime, intersectionality, containment

## Abstract

This study uses a historically informed lens of coloniality, bordering, and intersectionality to analyze maritime bordering discourses and practices that target seafarers recruited from the Global South who embody the border in their everyday lives. In seeking to explain the current context exemplified by the sacking of P&O Ferry workers and the recruitment of “foreign agency” crews in March 2022, the study foregrounds 19th- and 20th-century maritime bordering legislation on ships and onshore, focusing on public-/private-bordering partnerships between governments, shipping companies, and unions. Archival research on British Indian seafarers employed by P&O a century ago and analysis of contemporary media and political discourses relating to “foreign agency crews” are drawn on to consider the implications of earlier bordering discourses and practices for 21st-century British citizenship and belonging. Attending to imperial bordering regulations that created the racialized and class-defined labor category of *lascars* explains the “common sense” designations of seafarers recruited in the Global South and their families as potential “illegal migrants,” and in doing so, it constitutes the long history of the public/private partnerships that constitute the UK's “hostile environment” immigration policies.

## 1. Introduction

This special issue*, Bodies at Borders*, focuses on the objectification and containment of migrant bodies at border crossings. An immediate question is whether or not seafarers—who spend their working lives in transit at sea or contained at docksides and who are expected to return to their countries of origin between contracts—should be considered “migrants.” The negative answer to this question arises in the context of labor migration theory (Borovnik, 2004), particularly in relation to seafarers recruited from the Global South, whose passports limit their possibilities of settlement elsewhere and is made “common sense” in media and political discourse partially through the onshore invisibility of the everyday experiences of the racialized, class-defined and gendered hierarchies of seafaring life.

In this study, I explore historical contexts and practices of borders and borderings that have contributed to this 21st-century silencing discourse of global coloniality (Trouillot, 1996; Tlostanova and Mignolo, 2012). In doing so, I demonstrate how 19th- and 20th-century maritime and nationality legislation and bordering practices combined to prevent seafaring British Indian men racialized as *lascars* from settling in the UK and white settler colonies—an explicit aim being to prevent them from becoming legally settled “migrants.” I draw on, but cannot do justice to, wide-ranging research of colonial labor and maritime history which continues to contextualize and give voice to the experiences of seafarers recruited from Britain's empire (Tabili, 1994; Visram, 2002; Ahuja, 2006; Balachandran, 2012; Manjrekar, 2019). These, together with oral and family histories of Indian seafarers born in the first half of the 20th century collected by citizen historians, are the rare sources where the voices are heard of seafarers recruited under British colonial rule who eventually settled in the UK (Adams, 1987; Choudhury, 1993, 1995; Shakoor, 2018, 2020). Due to their confinement at sea, below deck in the engine rooms, exclusionary legislation, together with the (until recently) marginal academic interest in their globalizing significance (Balachandran, 2013), they remain outside most analyses of migration and border studies (cf. Popescu, 2012; Castles et al., 2014; El-Enany, 2020).

In centering the objectification and containment of seafarers recruited from the Global South, I use the key concepts of bordering and everyday bordering (Yuval-Davis et al., 2019). In doing so, I explore the interplays between neoliberal globalization and the coloniality of racialized national and international maritime employment practices, dockside accommodation policies, and laws that combine exclude, contain and control “foreign seafarers” imagined as suspected border crossers *ergo* “illegal migrants.” I show how the maritime bordering of bodies has proliferated from the days of sail through the era of steamships and continues to be central to national and global operations of neoliberal globalization. This article uses an intersectional lens in focusing on the classed and racialized bordering on board ships and at the littoral border crossing of the UK docks, the space between the competing jurisdictions of the ship and the land, and the material site of the discursive objectification and physical containment of racialized maritime laborers recruited overseas, who embody the British border. It demonstrates how maritime bordering laws of the British empire, enacted through partnerships between government, private companies, and individuals, constitute the long history of so-called “hostile environment” immigration policies where everyday bordering discourses and practices target differently situated working class, minoritized men and women (Wemyss, 2015; Yuval-Davis et al., 2018, 2019).

The article is divided into three sections. Section one sets out the theoretical framework through the illustrative example of the overnight dismissal by the DP World-owned P&O Ferries of 800 unionized “British” crews and their immediate replacement with low-paid “foreign” agency workers in March 2022. The theoretical and analytical framework draws together the concepts of global coloniality, neoliberal globalization, and bordering that I argue work together to create and maintain racialized, exclusionary, and hierarchical labor categories. The section focuses on parliamentary, union, employer, and media discourses about P&O Ferries and crews from 2022 to understand the coloniality of bordering experienced by seafarers explored in sections two and three. In seeking to understand the current context exemplified by P&O Ferries and DP World, section two attends to 19th- and 20th-century discriminatory colonial bordering employment laws experienced by British Indian seafarers employed on inferior contracts, which placed them in the racialized labor category of *lascars*. The section focuses on shifting bordering partnerships of the East India Company, British governments, and from the 1840s, the P&O company and their roles in creating, upholding, or sometimes challenging the exclusionary legislation and practices. Section three focuses on bordering onshore, reaching inland from the docks. I use the illustrative example from a century ago of a “hulk” reported as being used by the P&O Shipping Company to house British Indian seafarers in London's Royal Albert Docks. Discourses about the “hulk” and common lodging houses are discussed to explain historical practices of bordering where government, unions, and shipping companies partnered to control and contain British subjects categorized as *lascars*, imagined as potential “illegal” migrants. The conclusion brings together threads from these histories of public/private bordering practices *via* maritime legislation and the P&O shipping company's past and present discriminatory practices in the context of 21st-century neoliberal globalization to consider the implications of the continuing objectification and containment of racialized seafarers and these earlier bordering laws for 21st-century hierarchies of British citizenship and belonging.

## 2. Section one: P&O, coloniality, and bordering

On 17 March 2022, P&O Ferries (P&O) made 786 seafarers redundant, stating the necessity of improving business viability through a change to its crewing model from permanent to agency staff, many recruited from the Philippines. The seafarers were mostly British citizens living in the UK but employed under Jersey law working on ferries registered in Cyprus, Bermuda, and Barbados. P&O's strategy was to bypass legally binding consultation with the unions [Section 188 of the Trade Union and Labor Relations (Consolidation) Act 1992]. The employer calculated that they could afford any additional costs resulting from that breach of procedure by offering employees redundancy compensation above the legal requirements and incentivizing dismissed employees from costly disputes of the redundancies in the courts (Stones, 2022). P&O leaders had determined that no union would accept the new operating model and argued that any other option would result in P&O Ferries not being viable. The redundancies were announced with immediate effect *via* a pre-recorded video. Private security guards were employed to escort crew from ferries on the UK/Europe and UK/ Ireland routes. P&O Ferries management announced that the ships would remain in harbor for several days, while agency crew were brought in and trained. In the days that followed, news reports indicated that the new agency staff were predominantly “foreign,” employed on lower wages, and expected to live on board the ships for a 6-month period. While the voices of British employees and unions were, to different extents, present across various media, and conditions of “foreign” agency workers were described by others, the voices of agency workers themselves were absent (e.g., see BBC, 2022; Daily Mail, 2022; Hull Daily Mail, 2022; The Guardian, 2022; The Telegraph, 2022). I argue that the invisibility of the lives and voices of seafarers from the Global South, working from British ports, is constitutive of today's everyday bordering discourses that are rooted in colonial-era maritime everyday bordering legislation.

The words and actions of DP World, the global conglomerate owner of P&O Ferries, exemplify the view of neoliberalism as a form of government that sees democracy as an obstacle or even as an illegitimate intervention to the rule of the market (Brown, 2015). Soon after the sackings, the CEO of P&O Ferries, Peter Hebblethwaite, supported online by a representative of DP World, told a joint hearing of Parliament's transport and business committee that “there is absolutely no doubt that we were required to consult with the unions. We chose not to do so” (Topham, 2022). In this case, DP World was treating popular sovereignty, including the agreements reached between unions and governments, as inappropriate interference with the efficiency of the market. Nationally negotiated labor agreements are particularly challenging to global capital since the increased flexibility of labor, differential rates of pay, and heterogeneous labor markets have been integral to the expansion of global capitalism (Harvey, 1989; Brambilla et al., 2015). Different bordering legislation and practices have developed alongside the neoliberal restructuring of capitalism in ways that work to regulate labor (Mezzadra and Neilson, 2013). As others have argued, the processes of contemporary neoliberal globalization are context-specific and heterogeneous in effect, often contradictory and unstable (Ward and England, 2007; Kingfisher and Maskovsky, 2008), making it necessary to understand neoliberalism as it actually exists in its different manifestations, including through the various bordering processes that regulate capital and labor past and present (Yuval-Davis et al., 2019).

The absence of the voices of the seafarers recruited from the Global South in political and media discourses is constitutive of bordering processes that have contributed to their historical silencing in dominant narratives of British history. I explore in the following paragraphs how this 21st-century invisibility is rooted in legal and cultural colonial bordering processes that sought to ensure the containment of racialized seafarers in vessels at sea and in the liminal, littoral spaces of the docks in the metropole and white settler colonies. Like 19th-century seafarers, P&O Ferries agency staff embody the border, becoming identified as suspected illegal border crossers and as “not migrants.” Global coloniality frames and continues to form present-day state-bordering practices and everyday bordering processes of the UK. I bring the work of maritime historians (Balachandran, Ahuja, Ewald, and Tabili) into an analytical framework informed by the concepts of bordering and everyday bordering to evidence how colonial employment categories and related bordering discourses and immigration practices worked together over four centuries to exclude the seafarers recruited in the British Empire and thus to ensure that others recruited later from elsewhere in the Global South continue to be excluded from the UK.

Tlostanova and Mignolo (2012, p. 7) explain global coloniality as the “model of power relations that came into existence as a consequence of the Western imperial expansion but did not end with the official end of colonialism and colonial administrations.” While historical European colonialism is (mostly) past, the relations of coloniality endure. The power relations of global coloniality include historical cultural and labor relations together with knowledge production that both enables and restricts the ways differently situated people imagine their position in the world and their relationships with others. Twenty-first-century bordering processes targeted at seafarers are rooted in 19th-century laws that themselves evolved from 17th-century English legislation that all worked to include and exclude differently situated people in different times and spaces. Maritime and immigration bordering legislation, associated practices, and discourses created mobile labor categories that aimed to prevent working-class British Indian subjects from settling in the UK, producing and reproducing racialized hierarchies of Britishness and belonging. These relations of coloniality continue to circumscribe the lives of “foreign agency” seafarers, preventing Filipino seafarers from crossing the border when working in British waters and from settling in the UK.

Throughout the 20th and 21st centuries, governments in the Global South and North have been visibly strengthening state borders that were commonly created through European wars and colonial treaties. External walls or fences are constructed in parallel with increasing border checks at internal sites. While neoliberal globalization has been associated with the de-bordering of goods, financial services, and global elites, it has also been accompanied by re-bordering inside and outside of state territories in the name of securitization. State borders have always been created, reconstructed, and experienced in diverse ways, by differently situated people, at multiple levels and sites across time and space. They are intended to act as filters—permeable for those permitted to or able to cross them and impermeable to others (Yuval-Davis et al., 2019). Bordering processes constitute a principal organizing mechanism in constructing, maintaining, and controlling social and political order from local to global scales. van Houtum et al. (2005) notion of “b/ordering”—the interaction between the ordering of chaos and processes of border making—encapsulates the relationship between bordering and governance whereby b/ordering discourses and practices create and recreate categories of those who are included and those who are excluded from national collectivities. Processes of bordering always differentiate between “us” and “them,” those who are in and those who are out, those who are allowed to cross the borders, and those who are not. “Everyday bordering” refers to the everyday construction of *state* borders through ideology, cultural mediation, discourses, political institutions, attitudes, and everyday forms of transnationalism. In the UK, everyday bordering is integral to the government's “hostile environment” immigration regulations (Yuval-Davis et al., 2019). Through everyday bordering processes, state borders have moved into the center of political and social life, as citizens are obliged to check the immigration status of tenants, employees, and patients, for example, redefining contemporary notions of citizenship and belonging for racialized minorities and hegemonic majorities (Yuval-Davis et al., 2018). Thus, discourses and practices of borderings are situated and constituted through political negotiations and interwoven into the everyday intersectional encounters between differently situated individuals (Yuval-Davis et al., 2019). In present-day Britain, the UK, discourses and practices of everyday bordering materially and culturally reproduce exclusionary imaginations of Britishness and, as such, are enduring components of global coloniality that are experienced to different extents by differently situated people.

Immigration and nationality legislation have worked in bordering and racially ordering European nations over centuries of colonial expansion. Successive laws have created and policed borders that sought to maintain a global racialized order established by colonization. Empire-authored records were part of an ideology of containment that sought to convey that imperial control was effective in imposing racialized order onto a chaotic and transient situation (Goodall et al., 2008). Bordering discourses work in similar ways in the context of globalization, presenting an image of order being maintained in national imaginaries.

In the case of the UK, past and present legislation relating to Britain and its colonies have resulted in wealth accumulated globally being located within the borders of the UK. Immigration legislation has ensured that assets in the form of infrastructure, welfare provision, and future opportunities for citizens are inaccessible to most descendants of Britain's colonial subjects. In different times and colonial spaces, intentionally discriminatory bordering legislation has been made to appear “race-neutral” (El-Enany, 2020). Post-independence, bordering technologies such as those that constitute the hostile environment maintain the permeability in state borders for the citizens of Britain's white settler colonies while blocking citizens of Britain's African, Asian, and Caribbean colonies. While these arguments have been convincingly made elsewhere, the invisibility of the liminal working lives of seafarers has meant that the significance of maritime laws and practices of shipping companies to histories of bordering targeted at men recruited from the Global South are not well known. To contextualize 21st-century racialized maritime bordering processes, I now explore recent media and political discourses about the 2022 sackings of the P&O Ferry crew that have been, to some extent, formed through imperial-era discourses and practices of the P&O company.

### 2.1. P&O: Icon of empire

P&O is one of those businesses widely thought of as a British icon, but which has infact been chewed up by the machinery of globalization (Cumming, 2022).

The above statement, made in the features section of the *Daily Telegraph* soon after the sacking of the P&O Ferries' workers, was part of a dominant discourse that drew on notions of P&O as a national icon tied to Britain's historical greatness based on its sea power. While the past being referred to was obviously that of the British empire, the discourse avoided making direct links to Britain's imperial history. In this case, that avoidance was facilitated through the focus on UK-based ferries. Moreover, rather than being a victim of globalization, as this quote suggests, P&O, with its imperial roots and routes, was very much constitutive of neoliberal globalization's heterogeneous, shifting forms. As I argue in the following sections, the company's profits were boosted over 180 years through either lobbying government to regulate the labor market through specific bordering legislation, or in different circumstances, by bypassing bordering legislation.

The P&O name has existed since 1837 in various iterations and changing fortunes, buying up other companies and taking over different transoceanic routes. It was significant in servicing the British empire through its mail, cargo, and passenger steamships. The notion that P&O was a great national undertaking rather than simply a commercial steamer service was encouraged by the founding managing director, Arthur Anderson, who “positioned, promoted and politicized P&O as a company with name and influence” (Cox, 2022). In 1854, Anderson claimed:

[P&O] has now attained to a magnitude and national importance unprecedented in the annals of private maritime enterprise in this or any country of the world -a circumstance which I cannot help regarding with strong feelings of pride (Cox, 14 March 2022).

When P&O amalgamated with its main rival, the British India Steam Navigation, in 1914, the Peninsula and Orient Steam Navigation Company became the world's largest shipping conglomerate playing important roles in transporting food and troops in the First and Second World Wars. A 150th-anniversary publication referred to P&O as a “phenomenal company” that had diversified into enterprises well beyond shipping but remained anchored to traditions of trust, loyalty, service, and pride (Jack, 2022). As markets restructured in the context of neoliberal globalization, The P&O Steam Navigation Company bought various UK-based coastal shipping and ferry companies through the 1970s, 80s, and 90s, rebranding that part of the business as P&O Ferries. In 2000, the P&O cruise business was sold to Carnival, and in 2006, the rest of P&O, including P&O Ferries, was sold to Dubai World and in 2019 to the Dubai government-owned DP World transport and logistics conglomerate (Collard, 2021). At the time of the sackings, P&O Ferries accounted for ~15% of all cargo entering and leaving the UK, and DP World was a major investor in the multibillion-pound Solent and Thames Gateway freeport schemes near Southampton and London, respectively (Oliver and Cahill, 2022).

### 2.2. Racialized bordering discourses

Following the crew dismissals, political and media debates about the actions of P&O Ferries were constructed through a discourse that drew on related nationalist notions. These were as follows: first, of P&O as a British icon tied to imperial greatness and second, of British exceptionalism as an island, seagoing “race” threatened by globalization. That discourse was evident in journalist Cumming's later comment, “For an island race with sea in our veins, cross channel sailings are more than just transport” and that of travel writer, Adrian Bridges, quoted in the same article, “We are an island nation and going to sea has always been a huge part of our heritage and history. On a ferry we're connecting with who we are as a people” (Cumming, 2022).

Politicians and media commentators with different positionalities and political perspectives shared this discourse of P&O as a famous icon of the “island nation” in arguing for “decency,” “fair play,” and the need for “British crews.” In a special debate about the sackings (Hansard, 2022), the Conservative Party Transport Secretary drew on the imagery of national pride attached to the names of the foreign-owned and foreign-flagged ships to demonstrate his commitment to British workers.

To have a ship called *Spirit of Britain, Pride of Kent* or any other name that attaches it to this country when it does not have British workers would be completely wrong, and I will be calling on P&O to change the name of the ships (Grant Shapps M.P., UK Secretary of State for Transport *Hansard* 21/3/2022).

The Labor Party shadow minister used the same discourse when drawing on an imagined past of seafaring labor relations that, through the elision of colonial histories of legalized discrimination and conflict, I argue below, has contributed to the continuing exclusion of workers recruited from the Global South:

We are an island nation. British seafaring has been and is the envy of the world, and a sense of fair play and decency runs deep in this country: it is part of who we are. The action on Thursday was a straightforward assault on that tradition and on our values, so deeply entwined with our identity and synonymous with our global reputation. (Louise Haigh M.P., UK Shadow Secretary of State for Transport *Hansard* 21/3/2022).

In parallel with representing their British membership, union discourses contributed to the othering of agency crew members. On its website, the general secretary of the professional seafarer's union, Nautilus International, represented the sacking of crews by P&O Ferries as “a betrayal of British workers” (Nautilus International, 2022) and the leader of the National Union of Rail, Maritime and Transport (RMT) Workers was quoted using the dehumanizing word “import,” to refer to P&O's employment of “other” over “our” people:

We think they are importing Indian workers, Filipinos and Ukrainians at the moment to work on these vessels. That cannot be acceptable. We cannot dismiss our people to bring in other people on a discount rate (Daily Mail, 2022).

P&O Ferries' policy of replacing permanent British crews with cheaper foreign agency workers had started several years earlier. During the COVID-19 pandemic, the RMT had complained that the company was not working with the union to replace furloughed UK crews; instead, it was hiring agency crews from the Philippines below the UK minimum wage on the *Pride of Hull* (RMT, 2020). Karl Turner, the Hull MP, described agency crews who had earlier replaced British crews as going “to and from Rotterdam as prisoners in their crew cabins. Their terms of employment are appalling” (Hull Daily Mail, 2022). The on board containment and precarity of agency working conditions, although acknowledged by some commentators as reasons for the agency workers' compliance, silence, and invisibility, were not situated in the context of global coloniality in ways that would have challenged dominant discourses about the disputes.

In 2020, the P&O Ferries CEO justified low wages paid to Filipino crews in a discourse that obscures the context of global coloniality and neoliberal bordering that Mezzadra and Neilson (2013) have shown has created increasingly unequal labor markets. Through the silencing of colonial histories, that discourse is itself a constituent of 21st-century everyday bordering processes.

As a *family* brand, we take the welfare of *our* people seriously … all of *our* Filipino seafarers live on board on an “all found” basis during their tour of duty. This means that they benefit from food and accommodation free of charge and flights home to the Philippines are provided for in their contracts… The cash pay of *our* Filipino seafarers is closer to £4.50 per hour and substantially more when factoring in the free accommodation and food. Wages in shipping are unique. Yes—this basic figure is less than the UK National Minimum Wage, but that is completely irrelevant as the seafarers do not interact at all with the UK economy and, importantly, *they* consider this to be a fair wage. Indeed, their wage is 6.5 times higher than the minimum wage in the Philippines and twice as much as the average salary of anyone in *their* home country, where *they* spend *their* income (Hull Daily Mail, 2020; my emphasis).

In a dispute a year earlier, P&O Ferries had defended the 6-month rotation, whereby crews lived on board the ferries, as “standard industry practice in the maritime sector” and as “negotiated by the unions in their home countries” according to the “standards required by the International Transport Federation and the Maritime Labor Convention” (Hull Daily Mail, 2019). In section two, I show how the employment contracts that the P&O CEO refers to have roots in maritime bordering legislation that was continually adjusted in the days of imperial shipping to suit the interests of shipping companies, especially those of P&O and colonial governments. As well as the bordering work done by the contracts, the paternalistic language of a shared P&O family - where “our” workers are spoken for - echoes how the relationship between south Asian seafarers and European officers was constructed from the 17th century. It works as a bordering discourse in 2022 by excluding the voices of the workers (predominantly assumed to be men) and normalizes the neoliberal globalized work context positing that it is advantageous to be confined to a ship and separated from family for a 6-month period. In contrast, before that dispute, a white British male mechanic employed by P&O Ferries working the Dover—Calais route told me that he enjoyed his working pattern of 2 weeks on board ferries and 2 weeks off. He had to live on board for extended periods as it supported the 45-min change over time in the ports. However, he said he enjoyed the fortnight with his family, who all benefitted from a travel concession allowing them frequent holidays in Europe (interviewed in a Calais café in August 2014).

From a different perspective, the “island nation” trope was used at the time of the P&O sackings to argue for P&O Ferries and their crews as military reservists. *The Daily Telegraph* foregrounded security concerns about “foreign crews” through letters from Merchant Navy and Royal Navy officers:

Today, Britain's ocean-going fleet is almost entirely manned by foreigners, none of whom could be expected to fight and die for our country as did 35,000 merchant seamen during the Second World War. That P&O Ferries should sack its British seafarers does not come as a surprise. What is surprising is that the British Government should allow it given that ferries are the only means by which our soldiers can be delivered overseas, as they were in the Falkland Islands (Newton, 2022).

We are an island nation. As such we must ensure we have an adequate number of British officers and ratings available to man our merchant ships in both peacetime and wartime (Lang, 2022).

Both letters omitted that in the Second World War, a quarter of seafarers in the Merchant Navy were British Indian men, 6,600 of whom died in the conflict, which they were expected to support as subjects of empire (Visram, 2002, p. 347). The above-selected examples demonstrate how bordering discourses work to objectify and silence “foreign agency” workers recruited to work on P&O Ferries. While political and media discourses about the changes in the P&O labor strategy have included voices of British workers, beyond their union representatives and MPs, those of the “foreign” agency workers are harder to locate. Public debates have focused on the low pay, long working hours, lack of training, and relevant experience of agency workers in contrast to the long-term experience and redundancy compensation of the unionized “British seafarers.” In addition to the working conditions, the accommodation, rights to move across the border, and living conditions of agency staff are issues largely ignored.

To understand how contemporary bordering discourses, employment laws, immigration regulations, and other bordering practices work to reproduce exclusions of seafarers recruited in the Global South and related notions of white Britishness and belonging, it is essential to understand their colonial roots. While I cannot do justice here to the political and economic contestations that make up these complex histories, in the following two sections, I excavate illustrative examples of relations of global coloniality that have contributed to the framing of the parliamentary, shipping company, and media discourses that impact on the lives of agency workers employed by P&O Ferries.

## 3. Section two: Racialized bordering at sea

In seeking to understand the current bordering practices exemplified by the recruitment practices of P&O Ferries and DP World, I discuss selected historical material practices of bordering, including those supported by the P&O company in its earlier iterations, when its “flagships of Imperialism” supported by government mail subsidies and the opium trade was the largest British employer of Indian seafarers (Balachandran, 2012). As P&O was expanding toward becoming a global business, it worked with the British government to deny Indian seafarers equal working conditions and from settling in the UK.

In this section, I explore discriminatory colonial bordering laws experienced by British Indian seafarers employed on inferior contracts, which placed them in the racialized labor category of *lascar*. The classification of *lascar* secured through British parliamentary legislation and East India Company regulations and maintained through racializing discourses forced Indian seafarers into an employment category that ensured that they remained at the base of British Merchant Navy hierarchies (Visram, 2002). As well as denying *lascars* on board employment rights granted to white seafarers, the legislation excluded the *lascars*, who were British subjects, from the settlement in the UK. Public–private bordering partnerships between the British government, the East India Company, and later, the P&O, other shipping companies, and British unions worked throughout the period to ensure that south Asian seafarers embodied the border at sea, at the dockside, and inland.

During the 19th and 20th centuries, the complex array of bordering techniques grew out of the economic priorities of shipping companies that strove to keep costs down by maintaining a segmented, racialized labor market with Indian and African seafarers segregated in the bottom rungs of a rigid hierarchy (Tabili, 1994; Visram, 2002; Ahuja, 2006; Ewald, 2013a,b). These combined with bordering processes associated with racially exclusive immigration laws in Britain, North America, and Australia so that at different times and in different spaces, multiple states, and privately administered bordering techniques were put in place attempting to “contain” the itinerant seafarers at ports of departure in India, at sea and ports of entry. In the following paragraphs, I explore these borderingscapes (Yuval-Davis et al., 2019) to show how they worked together to ensure that working-class Indian seafarers faced considerable barriers in settling in Britain, thereby producing their invisibility in national narratives and normalizing the view demonstrated in the previous section, that seafarers recruited from the Global South are not entitled to settle in the UK with their families.

### 3.1. “*Lascar*” contracts

Legislation that discriminated against African and Asian sailors on British ships existed since the 17th century (Davis, 2012, p. 136; Ewald, 2013b, p. 277; Fisher, 2004, pp. 32–42; Tabili, 1994; Visram, 2002, pp. 16–20). The 1823 Merchant Shipping Act exemplifies most clearly how bordering legislation, discourses, and practices worked together to discriminate against British Indian seafarers, racialized as *lascars* on board, in the docks, and beyond the docked ships into local communities. The 1823 Act, not repealed until 1963, made official the employment category of *lascar*, which had been commonly used to label men from across south Asia employed on European-commanded ships. Seafarers recruited from very diverse areas were grouped into a single racialized category, employed on contracts that became known as “Lascar Articles.” These contracts confirmed that diverse Indian seafarers, when lumped into the racialized homogeneous employment category of *lascar* were not British subjects and could only be discharged and paid off in India. The contracts also defined their working conditions, compelling them to work in inferior conditions for less pay (detailed in the following section). *Lascar* became a term of racist abuse in the English maritime language, described as the mobile equivalent of *coolie* (Balachandran, 2012). Any *lascar* convicted of vagrancy in Britain had to be repatriated by the East India Company. Ship captains who failed to report the arrival of *lascars* in Britain faced a fine, one-third paid to the informer, and two-thirds paid for the prosecution and maintenance of the “illegal immigrant” seafarer (Fisher, 2004, p. 176). What had started as a response to Indian requests to return home became institutionalized as forced deportation, facilitated by citizen border guards, intended to prevent them from becoming legal migrants.

As well as preventing settlement, the enshrining of the inferior racialized category of *lascar* into British maritime law ensured that the legal minimum standard of accommodation for Indian workers on board ships, their contractual position, and diet scales lagged far behind those of white seafarers (Visram, 2002, pp. 18–33). By the 1840s, the increased imperial trade and lobbying from steam shipping companies, most notably P&O which was dependent on Indian labor, contributed to the British Parliament redefining *lascars* as “British.” This enabled ship owners to recruit more cheap labor, and P&O led the way in employing all-Indian crews on their steamships bound for Britain. With the increasing number of Indians arriving at British ports, the government passed further laws were passed denying British Indian seafarers settlement rights in Britain. The 1854 Merchant Shipping Act forced ship owners to pay a fine of £30 if any *lascar* was left behind in Britain. Numbers further increased after the 1869 opening of the Suez Canal (Tabili, 1994). The introduction of steamships created new segmented labor categories in the engine room, where half of all seafarers worked stoking the furnaces. From the 1850s, P&O began to recruit African crews from Indian Ocean ports. They were labeled as *seedies* and employed on inferior contracts in the stoke holes, where they overwhelmingly carried out the most dangerous role of trimming coal. The labor historian Janet Ewald argues that on P&O ships, African seafarers were segregated below the Indian seafarers into the “bottom layer of the racialized hierarchy” (Ewald, 2013b, p. 280). More *lascar* crew were employed for the equivalent number of Europeans; however, there remained a net gain for the shipping companies who argued that Indian and African workers were better suited to the excessive heat of the engine rooms. Arguments that Indian crews were unsuited to colder climates were used to justify paying them less despite their working on north Atlantic routes (Visram, 2002, pp. 55–56; Balachandran, 2016). As P&O grew, it became increasingly integral to the expanding empire, carrying its cargo, passengers, and government-subsidized mail between Indian, Asian, and Australian ports. The company became the largest single employer of Indian crews whom it recruited *via* networks reaching inland from its Bombay (Mumbai) terminus across the west of the subcontinent. In the east of India, The British India Steam Navigation Company (BISN) employed the second-highest number of Indian crewmen whom they recruited inland from networks centered in Calcutta (Kolkata) (Ewald, 2013b, p. 278). Notably, 100% of *lascar* crews were common on routes east of the Cape of Good Hope, and by the 1880s P&O was “almost wholly dependent” on them (Balachandran, 2016, p. 198).

In the later decades of the British Empire, there were continuing tensions between the “mobility” and “containment” of British Indian subjects who were moving around the empire as indentured laborers, military personnel, and seafarers (Ahuja, 2006). The seafarers were the most mobile and the hardest to monitor and contain. Steamships spent less time in dock than sailing vessels, and British Indian crew were not always allowed to land. The metropolitan response to their increased mobility reinforced the existing “tiered arrangements of racialized biopolitical borders” reaching into ships and foreign ports' (Balachandran, 2016, p. 188). The 1894 Merchant Shipping Act bound them to return to India by giving shipowners powers to place them on vessels heading back to India even without work, and Indian seafarers who deserted faced criminal prosecution (Fisher, 2004; Balachandran, 2012, p. 385; Visram, 2002, p. 56). Bordering technologies constructed to “contain” the Indian mobile labor force and prevent desertions and the settlement of working-class Indian men in the metropolis and white settler colonies mean that there is little material or discursive evidence of their time on land.

Multilayered partnerships between employers, unions, and compatriots made up the everyday practice of bordering legislation in different colonial contexts. At different periods, shipping companies made decisions about whom they employed based on contemporary racialized stereotypes and links with diverse local networks they had built up in specific localities. In the early 20th century P&O preferred Muslims from Punjab to work in the engines, deckhands from Gujarat, and Christian stewards from Goa, while the Clan steamship Company chose crew from Sylhet recruited in Kolkata (Ahuja, 2006, p. 130). Access to the ships and ensuing mobility reached inland to villages and households as influential crew members—the *serangs* (boatswain)—recruited *via* their own networks. *Serangs* also controlled the lives of seafarers on board through bonds of debt that reached back to villages. Their own dependence on the white officers and financial obligations meant that it was in their interests to ensure that Indian seafarers were kept under surveillance when anchored in docks and caught and punished if they attempted to cross the dockside border by deserting (Adams, 1987; Ahuja, 2006, p. 136; Balachandran, 2016, p. 198).

P&O took over its rival BISN in 1914. In the same year, Indian seafarers were estimated to number 51,000 men, forming 17.5% of the crew employed on British registered ships, servicing the Indian Ocean and international trade routes, including to Australia and Britain. The 1823 Indian Merchant Shipping Act, still in place, ensured that their conditions of labor remained inferior to that of British seafarers. Per month, Indian crew earned less than a quarter of that earned by white British crewmembers for equivalent work and were allocated just over half the living space of European sailors (Visram, 2002, pp. 54–55; Balachandran, 2016, p. 198). Indian men continued to be employed in large numbers during the First World War as white seafarers were recruited onto Royal Navy ships and, by 1919, formed 20% of the British maritime labor force, and by 1939, they made up over a quarter.

Indian seafarers resisted poor conditions and cruelty through deserting ships when possible while governments and shipping companies sought to prevent them from legally migrating to and settling in Britain or elsewhere in its white settler colonies (cf. Adams, 1987; Choudhury, 1993, 1995; Visram, 2002; Fisher, 2004; Balachandran, 2012, 2016; Manjrekar, 2019). In the following section, I explore the onshore bordering discourses and practices that sought to prevent their desertion, migration, and settlement.

## 4. Section three: Bordering seafarers onshore

In the days of sail, seafarers would spend several months in the port areas before obtaining a return voyage, and many became destitute and “illegal” on the streets of London. The East India Company (EIC) was obliged to house Indian seafarers in barracks near the ports or privately run boarding houses since they were prohibited from terminating their contracts anywhere outside of British India. Indian, African, Chinese, and Caribbean seafarers were targets of racist attacks and abuses throughout the 19th and early years of the 20th centuries (Visram, 2002; Fisher, 2004). In 1816, the EIC had recommended confining south Asian seafarers to “hulks” moored in the Thames to protect Britons from what they described as the “depravity” of their character (Fisher, 2004, pp. 173–174). The idea for the offshoring of racialized colonial subjects was a development of 18th-century government policy whereby old ships had been used to house convicts waiting to be transported or forced into hard labor locally. Floating on the Thames at Woolwich or elsewhere, hulks ensured the isolation of the prisoners from family and friends. The living conditions contributed to high levels of illness and death (PortCities, 2010). However, the EIC continued to confine Indian seafarers in barracks near the docks, and as I discuss later, the idea of using a hulk for housing re-emerged in reference to P&O in 1922.

In reference to the later era of steamships, Balachandran drew on Agamben (1995) in likening ships to “camps”—spaces of confinement and exception where states and private employers exercise “extraordinary power” over racialized seafarers (2016, p. 188). When ships run by P&O or their British rivals docked in ports across the British Empire, Indian seafarers were often not allowed to land, or if they did so, they were confined to warehouses or boarding houses discussed later. However, while state laws and economic disparities structured the lives of colonial subjects from recruitment in Indian villages through voyages and dockside barracks, the lived experiences of Indian seafarers resembled more the *campzenship* outlined by Sigona (2015) than the camp. In Sigona's 2015 conception, *campzenship* is a situated form of membership produced by the camp which accommodates the complexity of social relations in and around the camps through the resident's everyday interactions and practices with authorities and each other, reshaping rights, entitlements, and obligations. Rare oral histories of seafarers show how the on board voyages and dockside changeovers should be seen as elements of a continuum of littoral working lives (Wemyss, 2011). The Indian seafarers, although legally bound by their inferior contracts, negotiated their everyday lives in dialog with ships officers, *serangs*, accommodation officers, and boarding house owners, as well as networks that included compatriots and wives, parents, and extended family onshore on different continents or working on other ships (Adams, 1987; Gardezi, 1989; Choudhury, 1993, 1995).

What was referred to in shipping company and government discourses as “desertion” or “jumping ship” was effectively an attempt to cross the border and control the shipping companies. Seafarers outwitted officers when they moved illegally from ship to land or from the barracks or boarding houses where they were obliged to wait out their time. Especially during the war and post-war decades, they were actively recruited and employed illegally by onshore businesses. From the mid-1920s, despite the extension of maritime laws that required shipping companies to track down and prosecute British Indian “deserters,” only P&O did so because their trade, predominantly with Asia, depended to a greater extent on the low-waged “Lascar Articled” labor force. Other companies with more North Atlantic trade ignored desertions if they suited them economically (Balachandran, 2012, pp. 181–184).

In addition to the financial interests of rival shipping companies, the racially discriminatory maritime and immigration laws, and the different backgrounds of the crews themselves, the bordering processes that limited the mobility and strengthened the containment of colonial crews were contingent on the politics of the British seafaring unions locally and globally (Balachandran, 2016, p. 196). The public–private bordering partnerships were, for different reasons, supported by white seafarers' unions. Throughout the years of the empire, stereotyped views of Indian seafarers were mobilized by ship owners, captains, and unions. Their abstention from alcohol was seen as an advantage by officers, contrasted with what they saw as the “drunkenness and absence without leave” of white employees. The racialized inferior category of *lascar* was associated with lacking masculinity and initiative. Constructions of the “docility” were produced through the racialized political and economic relations of empire and the domination on the ship and on land where the ship's officers were empowered to wield control over every aspect of their lives. These racist constructions were used by shipping companies to justify their inferior conditions of employment (Visram, 2002; Ahuja, 2006). Even the pensions of Indian seafarers were bordered in favor of British residents. They did not receive pensions because although ship owners, under the 1911 National Insurance Act, were obliged to contribute to a pension fund for “*lascars*,” seafarers who did not live in Britain were excluded from receiving the pension. Instead, white ex-seamen benefitted from the payments (Visram, 2002, pp. 55 and 225–226).

Before the First World War, British seafarer's unions supported a range of bordering techniques to prevent the employment of “foreign labor.” Union leaders used racializing and emotive language in their opposition to the recruitment of un-unionized colonial labor whom they represented as depriving white seafarers of work and better conditions. While those racialized as Chinese were the main target, other groups of racialized seafarers were included in the vilification and demands. Using a discourse of on board safety and “race neutrality,” the president of the National Sailors' and Firemen's Union (NSFU) demanded that Indian and Chinese seafarers should be fluent in English. This was clearly not needed since the labor categories on board steamships, described in the previous section, were well known to be segmented and controlled by the *serang* intermediaries. Racialized seafarers were regularly targeted as being the cause of the bad conditions of white seafarers. In 1911, the chairman of the Clyde branch of the National Transport Workers' Federation (NTWF) argued that Chinese and Indian seafarers “lowered the standard of life for white men” struggled for by the unions and threatened that it the “Chinese Invasion” continued, “the workers would have one of the biggest fights that the country has ever known.” In the same year, while in London, NTWF leader Ben Tillett complained that the shipping companies had “engaged all possible Asiatics and foreigners including negroes” forcing white crews out (Visram, 2002, pp. 57–58). By 1913, the NFSU leadership had “abandoned any pretense of inter-racial solidarity” to campaign for the complete exclusion of Chinese seafarers from British ships (Tabili, 1994, p. 88). In the case of Indian seafarers, the shipping companies wanted to avoid aggravating white seafarer's unions in both the UK and Australia where at a different time the unions had taken P&O to court over employing “colored” seamen. Whether constructing Indian seafarers as threats or victims, the actions of the white unions supported their respective governments' efforts to stop working-class Indian men from coming ashore and settling in the growing cosmopolitan dockside communities (Goodall et al., 2008, pp. 56–57).

### 4.1. Negotiating bordering at the dockside

The memoirs of Dada Amir Haider Khan, who worked as a seafarer during the First World War, counter the one-dimensional constructions of Indian men who were compelled to stay on board or in approved lodgings when they arrived in British ports. Arriving on the P&O Steamship, the SS Khiva in the Royal Victoria Docks in the winter of 1917–1918 Khan wrote of leaving the ship and docks to visit acquaintances who were living and working onshore. In doing this, Khan and his friends were “illegal border-crossers.” During and after the First World War, Indian seafarers were recruited and illegally employed by businesses such as Tate&Lyle located near the docks. Khan also gives an idea of the P&O accommodation and security arrangements for Indian seafarers:

I encountered a former shipmate of my senior brother whom I knew from Bombay. He was residing in a working class locality of London where he was employed in some factory. A few times he took me to his lodging house and other places where the working people lived … After taking some additional men from the reserve which the P&O kept near the docks, and having our photographs taken for identification cards, the S.S. Khiva crawled out of her mooring place in January 1918 (Gardezi, 1989, p. 120).

Khan deserted the ship at the end of that voyage in New York, quickly obtained naturalization papers, and got recruited onto an American ship with better conditions and freedom to leave the vessel when it docked in Liverpool 2 months later. However, he remained conscious of the risks faced by Indian seafarers crossing the border when he visited the P&O ship he had previously deserted when it returned to New York:

We purchased some fruit and accompanied these men to visit the rest of our shipmates and friends … it was daring on our part to board a ship that we had escaped from illegally a short time earlier. But the *serang* would not have detained us forcibly in the presence of so many of our friends (Gardezi, 1989, p. 133).

Khan's memoirs hint at the interactions and negotiations among seafarers, *serang*, white officers, and dockside populations that are invisible in most official and unofficial archived material. He recalled that men on board had been able to tell people from his village and his mother that he was in good condition after deserting (*ibid*). Imperial bordering laws were not the impermeable mechanisms of control represented by governments.

In 1919, riots in port areas of the UK were started by local white populations who attacked people and property of the mixed neighborhoods, blaming African and Asian laborers for the lack of employment during the economic downturn (Tabili, 1994). The government's response was the 1919 Aliens Restriction (Amendment) Act that ordered preference be given to British crews, assumed to be white, and the deportation of “destitute colored seamen.” Despite being officially categorized as British subjects and not “Aliens” Indian seafarers often had no documentary proof of their status, and many were deported alongside seafarers from different areas of Africa and the Caribbean (Ahuja, 2006).

During this period, onshore accommodation of seafarers was associated with an array of state-bordering practices. The case of the 1922 surprise inspection of official and unofficial lodgings in dock areas of east London illustrates how these practices were aimed at preventing desertion, avoiding racial conflict that would have a negative impact on public opinion in India (thus preserving the ideology of imperial superiority), protecting profits of the shipping companies, or a combination of all of the above (Tabili, 1994, pp. 59–65; Balachandran, 2016, p. 198). The inspection party consisted of MPs led by Earl Winterton (the Parliamentary Secretary to the India Office), a missionary employed by the Port of London, and representatives from the LCC. The inspection included the relatively expensive “racially segregated” Strangers Home for Asiatics, Africans, and South Sea Islanders (favored by the government representatives and the LCC); the house of Choy Sing in Poplar and other unlicensed “common lodging houses” suspected of housing Indian seafarers with Chinese seafarers; and the P&O managed “hulk” in the Royal Albert Dock (possibly the place where the P&O kept their “reserve” referred to by Dada Amir Haider Khan earlier). Unlike the common lodging houses, which the inspection party saw as actively encouraging seafarers to find work onshore or enlist elsewhere on better-paid British Articles, the isolated dockside location of the P&O “hulk” made desertions hard. The location also meant that while the London County Council (LCC) had the authority to inspect and license boarding houses, they had no authority over the “hulk.” This was the main concern of Dr. Kay Menzies of the London County Council (LCC) Health Inspectorate:

I have reason to believe that this accommodation consists of an old hulk in the Royal Albert Docks. It is under the supervision of the Port of London Sanitary Authority and is therefore outside our jurisdiction and cannot be inspected by any member of our staff. I am given to understand therefore that this hulk is an “abomination” and a byword in the Dock neighborhood for filthiness and unsuitability… [L/E/7/1152, 1922, Kay Menzies to Cobb, 15 June 1922].

P&O gave permission for the party to visit the “hulk,” after which it was referred to as a shed in the resulting report and communication. It was reported that P&O called it a *godown* (a word used for a warehouse in parts of Asia)—suggesting perhaps that its isolation had led to the rumor of a floating “hulk” (Winterton to Peel 4 December 1922 L/E/7/1152). Floating or not, the inspection report confirmed that conditions were “unsatisfactory in every detail,” dirty, badly heated, no proper cooking arrangements, and insufficient space (Segrave Report, 1 December 1922 L/E/7/1152). Not wanting to antagonize the shipping company, the India Office sent P&O private communications about the “disgraceful” quarters, to which its directors responded that they were already planning to demolish them (Communications between Peel and Shaw 1–11 January 1923 L/E/7/1152).

A conference to discuss the government's response to a forthcoming parliamentary question about “*lascar* accommodation” following the inspection made suggestions that aimed to develop the bordering roles and partnerships between owners of lodging houses, the LCC, the India Office, and shipping companies. The first suggestion to prevent desertions was that the LCC should introduce regulations to compel lodging housekeepers to report to the India Office within 24 h of the arrival of any Indian seafarer, their name, the name of their ship, and the reason for leaving their ship. Another was to follow Australia and Canada in legislating for shipowners to be fined for every Indian desertion. A further suggestion to avoid “racial disturbances” was to house Indian seafarers separately from others (Conference on Lascar Accommodation, 8 December 1922. L/E/7/1152).

Bordering discourses of Conservative and Labor MPs who took part in the parliamentary discussion on *lascar* accommodation also worked to exclude and silence the experiences of Indian seafarers in the UK. In responding to the question on *lascar* accommodation, the Conservative MP, Earl Winterton, said that he had been part of the inspection, but he shared none of the details that would have alerted others to the appalling living conditions of working-class British Indian men. He said that he “had come to the conclusion that there is room for considerable improvement in certain cases” (Hansard, 1922). Manny Shinwell, Labour MP, and former activist in the British Seafarers' Union (BSU) showed no interest in knowing more about those conditions as he switched the focus to the accommodation of white seafarers and ways to prevent British Indian seafarers from landing in Britain:

Will the Department at the same time inquire into the housing accommodation provided for white seamen in various ports of this country? Cannot steps be taken to prevent crews being shipped on vessels to be paid off at British ports so that they shall not be discharged in this country? (Hansard, 1922).

In the following decade, the situation for racialized seafarers deteriorated further with legislation including the explicitly racist 1925 Colored Alien Seamen Order that required “colored seamen” to register with the police and be deported if “destitute.” African and Caribbean men, Goan Christian seafarers who were not categorized as British Indian and British Indian crew without papers that proved their status as British subjects were deported (Ahuja, 2006). However, many men successfully escaped the ships and “Lascar Articles,” using growing Indian networks in port cities to find work on land and ways to get employed back onto ships on British Articles, giving them better conditions than the European crew. During the Second World War, British Indians continued to be the subjects of surveillance as both state and non-state actors took on bordering roles around the docks and inland. The National Union of Seamen and port authorities “sought closer watch on Asian boarding house keepers to check desertions” and “any constable or military officer” was empowered to “arrest an Indian on mere suspicion of desertion” (Balachandran, 2012, pp. 186–187, see also Visram, 2002; Ahuja, 2006). Bordering discourses and practices meant that the UK border continued to be wherever an Indian seafarer was onshore. In the context of 21st-century global coloniality, their male and female descendants, together with seafarers recruited elsewhere in the Global South, remain targets of embodied bordering discourses and practices.

## 5. Conclusion: Embodied bordering

This study is a partial response to the question of whether seafarers, recruited from the Global South and working between British and European ports but prohibited from settling in the UK, should be considered “migrants.” I have argued that the normalization of the view that they are not “migrants” is due to the accumulation, since the early years of British colonialism, of racially discriminatory maritime legislation, everyday bordering practices, and discourses that forced racialized seafarers to embody the border.

Throughout the 19th and early 20th century, British imperial legislation aimed to make British borders differentially permeable to British subjects and those categorized as “aliens.” Indian men, racialized and class-defined through the labor category of *lascar* were targeted as undesirable migrants. The British border was never impermeable to British Indian working-class seafarers. However, by making it illegal to leave their ships and compelling them to initially live and work without documents, the bordering legislation forced them to hide from officials and private individuals, further making them and their families invisible as migrants. Immigration laws combined with maritime legislation produced and maintained the cultural whiteness of the metropole and settler colonies. In dialog with the legislation, bordering discourses worked to exclude and silence the voices of men recruited from coastal and inland colonized India but whose lives were spent crossing oceans between empire ports and elsewhere. More invisible still were the lives and voices of their families in India, while ideologies of racial purity stigmatized their families in Britain.

Bordering legislation and practices from 150 years ago constitute the power relations of coloniality that structure lives today. The proliferation of the differential bordering of bodies continues to be central to national and global operations of neoliberal globalization. In 2022, dominant political and media discourses mostly ignore the everyday lives of agency seafarers recruited from the Global South. However, the contracts they work under construct them, including those working on P&O Ferries in British waters, as potential “illegal migrants.” Empire-era bordering legislation and bordering discourses have normalized the conditions of living unseen and unheard on board for 6-month stretches, on low pay, away from families, and yet constructed as part of the “family” of the DT World conglomerate. As in the case of British Indian seafarers, the racialization of “foreign agency” crew ensures that they embody the border on sea and onshore.

In centering racialized bordering discourses and partnerships between government and private companies on ships, at the border-crossing spaces of UK docks and onshore, I am arguing for a deeper awareness of and further sociological research into the histories of marginalization, objectification, and physical containment of racialized maritime laborers. Along with swathes of bordering legislation across the empire, the public/private partnerships between the colonial governments and shipping companies constitute the long history of the UK's so-called “hostile environment” immigration policies whereby everyday bordering discourses and practices that have drawn ordinary citizens into border-guard roles, continue to target racialized working-class men and women (Yuval-Davis et al., 2019). In the present-day UK, everyday bordering materially and culturally reproduces exclusionary imaginations of Britishness and, as such, are enduring components of global coloniality.

## Data availability statement

Publicly available datasets were analyzed in this study. This data can be found here: https://www.bl.uk/subjects/south-asia.

## Author contributions

The author confirms being the sole contributor of this work and has approved it for publication.
